# Curvilinear Sub-Resolution Assist Feature Placement Through a Data-Driven U-Net Model

**DOI:** 10.3390/mi16111229

**Published:** 2025-10-29

**Authors:** Jiale Liu, Wenjing He, Wenhao Ding, Yuhang Wang, Yijiang Shen

**Affiliations:** School of Automation, Guangdong University of Technology, Mega Education Center South, Guangzhou 510006, China; 2112304033@mail2.gdut.edu.cn (J.L.); 2112304195@mail2.gdut.edu.cn (W.H.); dingwenhao@mails.gdut.edu.cn (W.D.); wangyuhang2777@163.com (Y.W.)

**Keywords:** inverse lithography technology, SRAFs optimization, level-set method, convolutional neural network (CNN), optical proximity correction, U-Net, lithography model, deep learning, computational lithography, mask optimization

## Abstract

In advanced semiconductor manufacturing, computational lithography, particularly sub-resolution assist features (SRAFs), is crucial for enhancing the process window. However, conventional SRAF placement methodologies are hampered by a critical trade-off between speed and pattern fidelity, and they largely fail to optimize the complex, curvilinear layouts essential for advanced nodes. This study develops a deep learning framework to replace and drastically accelerate the optical refinement of SRAF shapes. We established a large-scale dataset with coarse, binarized SRAF patterns as inputs. Ground-truth labels were generated via an Level-Set Method (LSM) optimized purely for optical performance. A U-Net convolutional neural network was then trained to learn the mapping from the coarse inputs to the optically optimized outputs. Experimental results demonstrate a dual benefit: the model provides a multi-order-of-magnitude acceleration over traditional CPU-based methods and is significantly faster than modern GPU-accelerated algorithms while achieving a final pattern fidelity highly comparable to the computationally expensive LSM. The U-Net-generated SRAFs exhibit high fidelity to the ground-truth layouts and comparable optical performance. Our findings demonstrate that a data-driven surrogate can serve as an effective alternative to traditional algorithms for SRAF optical refinement. This represents a promising approach to mitigating computational costs in mask synthesis and provides a solid foundation for future integrated optimization solutions.

## 1. Introduction

The steady advancement of semiconductor technology, characterized by Moore’s Law [1], has pushed integrated circuits to the deep nanometer scale, posing significant challenges to manufacturing fidelity. At the heart of these challenges lies the fundamental physical limit of optical diffraction. To surmount this barrier, computational lithography has emerged as an indispensable enabling technology. Central to this field is a suite of resolution enhancement technologies (RETs) [2,3,4,5,6,7], wherein optical proximity correction (OPC) [6,8] and the strategic placement of sub-resolution assist features (SRAFs) [9] are principal strategies. These SRAFs—non-printing patterns on the mask—are essential for enlarging the process window (PW) [10] and ensuring robust, high-yield manufacturing. Consequently, the optimal placement and design of SRAFs has become one of the most consequential, and computationally demanding, tasks in modern mask synthesis.

To address the demanding task of SRAF placement, methodologies have historically been categorized as either rule-based or model-based. Early approaches were predominantly rule-based, with pioneering work by Liebmann et al. [11] establishing a framework that leverages geometric rule decks to rapidly place assist features. A critical limitation of these methods, however, is that their rule decks are fundamentally designed for rectilinear geometries, thus restricting them to placing only simple, straight-line SRAFs. While computationally efficient, this inherent inflexibility leads to sub-optimal performance and poor adaptability to the increasingly complex and curvilinear layouts of advanced nodes, rendering them inadequate for modern lithography challenges.This inadequacy prompted a paradigm shift toward model-based techniques, which have since become the state of the art. The most holistic of these is inverse lithography technology (ILT) [12,13,14,15,16]. Crucially, ILT directly overcomes the limitations of rule-based methods by treating the mask as a free-form canvas, enabling the generation of complex, curvilinear SRAFs. It operates by seeking to solve the inverse problem: computationally determining the optimal photomask required to produce a given target wafer pattern. Although theoretically powerful, a full, pixel-based implementation of ILT is often computationally intractable due to the vastness of the solution space. Consequently, a prominent branch of ILT makes the problem more manageable by constraining the optimization to feature boundaries, frequently utilizing mathematical frameworks like the Level-Set Method (LSM) [14,17,18,19]. However, even this boundary-based approach suffers from a significant computational burden. Achieving a high-fidelity solution still requires thousands of resource-intensive simulation-and-update iterations, creating a severe bottleneck for any large-scale application. Therefore, a critical trade-off between optimization quality and computational efficiency persists. A significant research gap exists for a new paradigm that can achieve the high fidelity of model-based methods like ILT without their prohibitive computational expense. This challenge has motivated the exploration of data-driven approaches, particularly deep learning, as a promising avenue to bridge this gap.

**Figure 1:**
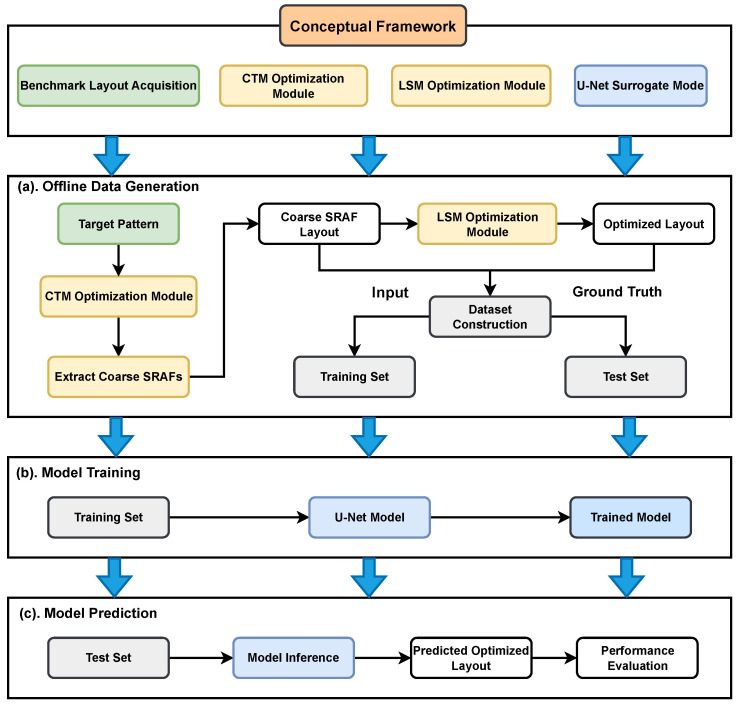
The overall framework.

To bridge the aforementioned accuracy-efficiency gap, our proposed methodology is realized through a data-driven, two-stage process. In the first stage—offline data generation—we establish a high-quality dataset. Initial coarse SRAF patterns are derived from a continuous transmission mask (CTM) [20,21], and these serve as the inputs for our network. The corresponding ground-truth labels are then produced by optimizing these coarse patterns with a conventional LSM. In the second stage—model training and prediction—a U-Net architecture is trained to directly learn the transformation from the coarse SRAFs to the LSM-optimized layouts. The technical route is shown in Figure 1. In practice, this trained model entirely bypasses the iterative LSM, offering a solution that is orders of magnitude faster and, as our results demonstrate, achieves a higher degree of optimization.

## 2. Physics-Based Pipeline for Dataset Generation

This chapter describes the ’offline’ data generation stage of our methodology, a process designed to construct the high-quality dataset for training our U-Net surrogate model. The workflow is presented in three subsequent sections: first, the underlying lithography simulation model is established; second, the procedure for generating the coarse SRAF inputs is described; and third, the LSM used for creating the ground-truth targets is elaborated.

### 2.1. Lithography Simulation Model

**Figure 2:**
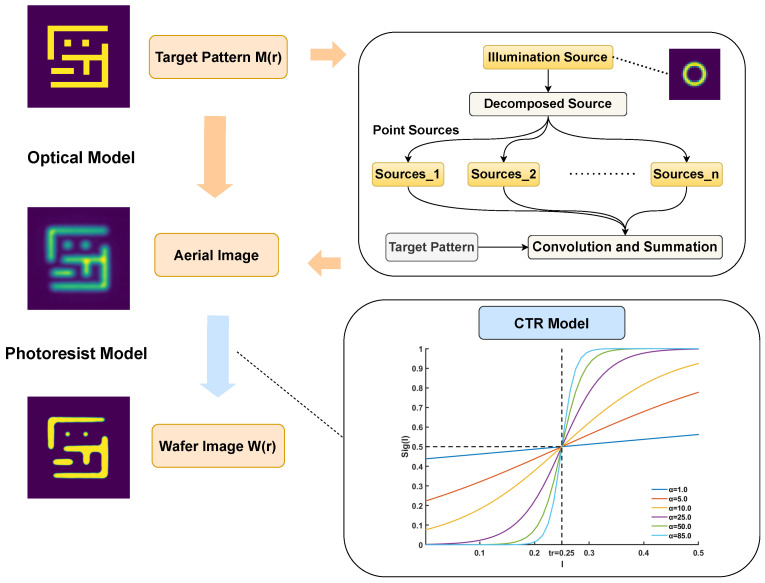
Flowchart of the lithography simulation model.

Any model-based lithography optimization relies on an accurate forward lithography model. This model, which we denote as an operator Γ, provides a mathematical abstraction of the physical process, simulating the final wafer pattern W(r) that results from a given input mask M(r). It consists of two sequential components: an optical model and a photoresist model. It is shown in Figure 2.

The optical model first describes the propagation of light from a given illumination source through the projection system. Under the Sum-of-Coherent-Systems (SOCS) approximation, the partially coherent illumination source was decomposed into a set of mutually incoherent but individually coherent point sources. The final aerial image I(r) [22] is the weighted sum of the intensities produced by each of these coherent systems:

(1) $$I\left(r\right)=\sum_{k=1}^{N}\mu_{k}{\left|\left(h_{k}⊗M\left(r\right)\right)\right|}^{2},$$

where $\mu_{k}$ represents the intensity of the *k*-th source point, and $h_{k}$ is the corresponding coherent point spread function, or kernel, of the optical system.

Subsequently, the photoresist model translates this aerial image into a physical resist pattern. For this study, we employed a Constant Threshold Resist (CTR) model [8] for a positive-tone resist. In this model, resist was removed wherever the light intensity I(r) exceeds a specific dose threshold, $I_{\mathrm{th}}$, and remains where it does not. The resulting binary wafer pattern can be represented by a step function H(I):

(2) $$H\left(I\right)=\left\{\begin{array}{cc} 0, & \mathrm{if}\;I<I_{\mathrm{th}}\\ 1, & \mathrm{if}\;I\geq I_{\mathrm{th}} \end{array}\right..$$

The non-differentiable nature of the step function, however, makes it ill-suited for the gradient-based optimization algorithms employed in the LSM. To create a differentiable forward model suitable for such optimization, we therefore approximate the hard-thresholding behavior of the CTR model with a continuous sigmoid function, sig(I(r)). To correctly model the response of a positive resist (where high intensity yields a value of 0), this function is defined as follows:

(3) $$\mathrm{sig}\left(I\left(r\right)\right)=\frac{1}{1+e^{-a(I\left(r\right)-I_{\mathrm{th}})}},$$

where the parameter *a* controls the steepness of the transition, thereby modeling the resist contrast.

Therefore, the complete forward model, predicting the final wafer image W(r), can be expressed by combining these steps:

(4) $$W\left(r\right)=\Gamma \left\{M\left(r\right)\right\}=\mathrm{sig}\left(I(r)\right)=\mathrm{sig}\left(\sum_{k=1}^{N}\mu_{k}{\left|\left(h_{k}⊗M\right)\left(r\right)\right|}^{2}\right).$$

### 2.2. CTM-Based Synthesis of Coarse SRAF Layouts

While the forward model from Section 2.1 simulates on-wafer results, the inherent low-pass filtering of optical systems causes significant pattern distortion if the target layout is used directly as the mask. To counteract this, inverse lithography technology (ILT) was employed to solve for an optimal mask. Our work utilized a specific gradient-based ILT implementation, CTM optimization, to generate a high-fidelity grayscale mask. The optimization was guided by minimizing the Pattern Error (PE), the squared Frobenius norm between the simulated wafer image W(r) and the target $W^{*}$. Although other metrics like Edge Placement Error (EPE) [23,24] and Normalized Image Log-Slope (NILS) [25] are vital for final verification, PE was selected as the cost function for its holistic, pixel-wise nature and, crucially, its differentiability, which is essential for the gradient-descent algorithm at the core of the CTM process. This transforms the optimization problem into minimizing the following cost function:

(5) $$F\left(M\left(r\right)\right)={∥W\left(r\right)-W^{*}∥}_{2}^{2}.$$

However, a direct optimization of the binary mask (M(r)∈{0,1}) is a challenging non-linear, discrete problem. To make this problem tractable for gradient-based methods, we employed the Continuous-Tone Mask (CTM) parameterization technique. This approach represents the mask transmittance using a continuous variable θ:

(6) $$M\left(\theta \right)=\frac{1+\cos(\theta )}{2}.$$

This cosine transformation effectively converts the constrained binary problem into an unconstrained, differentiable optimization with respect to θ. By substituting the complete forward model (including the sigmoid resist approximation from Section 2.1) and the mask parameterization into the PE cost function, we can define the final unconstrained objective function purely in terms of θ:

(7) $$F\left(\theta \right)=\sum_{k=1}^{N}{\left(W_{k}^{*}-\mathrm{sig}\left(\sum_{j=1}^{N}\mu_{j}{\left|{\left(h_{j}⊗\frac{1+\cos(\theta )}{2}\right)}_{k}\right|}^{2}\right)\right)}^{2}.$$

To solve this optimization problem, we applied the gradient descent method. In each iteration, the parameter vector θ was updated in the direction opposite to the gradient of the objective function, $\nabla_{\theta }F$. The update rule is given by the following:

(8) $$\theta_{t+1}=\theta_{t}-s\;\cdot \;\nabla_{\theta }F\left(\theta_{t}\right),$$

Here, *t* is the iteration number, and *s* is the learning rate that controls the step size. This iterative process continues until the cost function F(θ) converges, yielding the final high-fidelity grayscale mask, which is rich with nascent SRAF structures and serves as the ideal foundation for the subsequent binarization process.

**Figure 3:**
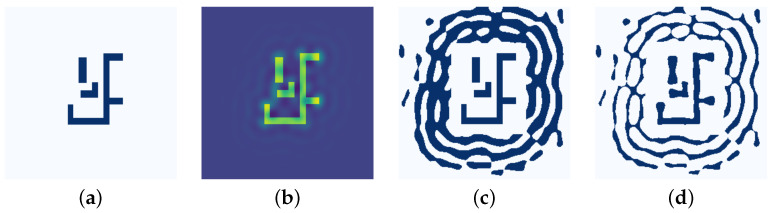
Illustration of the data generation pipeline: (**a**) initial target pattern, (**b**) the synthesized CTM, (**c**) coarse SRAF pattern after binarization (input x), (**d**) final LSM-optimized layout (ground-truth y).

The high-fidelity grayscale mask produced by the preceding CTM optimization serves as a rich, continuous-tone foundation for pattern extraction. The goal of our synthesis algorithm is to intelligently interpret this continuous-tone data, extracting the salient SRAF information embedded within it while judiciously discarding noise. This was achieved by first setting an adaptive binarization threshold based on the intensity of local maxima. The resulting pattern was then topologically refined through a filtering process that removes isolated regions smaller than a predefined area. Finally, to preserve design integrity, the original target pattern was explicitly reinstated, overwriting any generated SRAFs in its immediate vicinity. This entire procedure yields a coarse SRAF pattern, providing a structurally sound, albeit geometrically unrefined, input (*x*) for the U-Net model. As illustrated in Figure 3, the initial target pattern (a) was first transformed into an intermediate grayscale mask (b) via CTM optimization, from which the final coarse SRAF pattern (c) was then extracted.

### 2.3. LSM-Based Generation of Ground-Truth Layouts

The coarse SRAF pattern derived previously provides the input (*x*) for our supervised learning framework. To create the corresponding high-fidelity ground-truth labels (*y*), we employed the LSM to iteratively refine each coarse pattern into an ideal target layout. The LSM is a powerful numerical technique for evolving complex shapes, where the SRAF boundaries are represented implicitly as the zero-contour of a level-set function, Φ(r,t). The evolution of this function is governed by the Hamilton–Jacobi partial differential equation:

(9) $$\frac{\partial \phi }{\partial t}+V_{n}\left|\nabla \phi \right|=0,$$

The velocity field, $V_{n}$, which drives this evolution, was engineered to minimize the PE cost function (*F*) while ensuring boundary smoothness. It comprises two primary components: a data-driven term and a regularization term. The data-driven term, derived from the negative functional derivative of the cost function (−∇F), steers the boundaries toward an optimal optical solution. The regularization term, proportional to the boundary’s mean curvature (κ), prevents the formation of noisy, non-manufacturable features. The complete velocity is thus defined as follows:

(10) $$V_{n}=-\nabla F+b\;\cdot \;\kappa ,$$

where *b* is a small weighting coefficient.

To solve this partial differential equation numerically, we utilized a high-order Weighted Essentially Non-Oscillatory (WENO) scheme to accurately compute the spatial derivatives required for the velocity terms. The function was then updated iteratively using a Forward Euler time-stepping scheme. In each iteration *t*, the level-set function was updated according to the discretized rule:

(11) $$\phi_{t+1}=\phi_{t}-\Delta t\;\cdot \;V_{n}\left|\nabla \phi_{t}\right|,$$

where the time step Δt is dynamically adapted based on the CFL condition to ensure stability.

This iterative process continues until the PE cost function converges, yielding the final, high-fidelity optimized SRAF layout. As illustrated in Figure 3, the data generation pipeline creates the input–output pairs for training. First, the initial target pattern (a) is transformed via CTM optimization into an intermediate grayscale mask (b), from which the coarse SRAF pattern (c) is extracted. This pattern (c) serves as the direct input (*x*) for the U-Net model. It is then paired with its corresponding ground truth (*y*), which is the final, LSM-optimized layout shown in panel (d).

## 3. U-Net Surrogate Model Framework

### 3.1. U-Net as a Surrogate Model

While the LSM detailed in the previous chapter yields high-fidelity SRAF layouts, its iterative, simulation-intensive nature renders it computationally prohibitive for practical, large-scale applications. To surmount this critical efficiency bottleneck, we propose a paradigm shift from direct physical optimization to a data-driven deep learning approach, employing a U-Net as a surrogate model. The U-Net architecture [26,27] is exceptionally well-suited for this task due to its proven efficacy in image-to-image translation problems. Its distinctive encoder–decoder structure, augmented with skip connections, enables the network to learn the complex, non-linear mapping from a coarse SRAF input to its LSM-optimized counterpart. Crucially, by framing the problem as a mapping from a coarse SRAF to a refined one, we are not asking the network to generate patterns from scratch. Instead, we are teaching it a more constrained and stable refinement task. The coarse SRAF provides a strong topological prior, guiding the network to focus on the intricate optimization of feature boundaries rather than their initial placement. The ultimate objective of this framework is to replace the slow, iterative optimization with a single, rapid inference step, thereby achieving high-accuracy SRAF generation at a fraction of the computational cost.

### 3.2. U-Net Architecture

**Figure 4:**
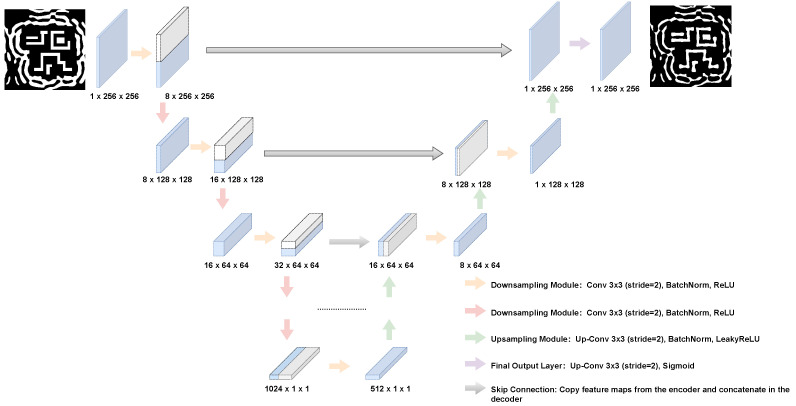
The U-Net architecture.

The architecture of our surrogate model is based on the U-Net, a fully convolutional network renowned for its efficacy in dense, pixel-wise prediction tasks. As illustrated in Figure 4, the network features a symmetric contracting (encoder) and expansive (decoder) path. The encoder progressively extracts hierarchical features and reduces spatial dimensions through a series of modules, each comprising a strided 3 × 3 convolution, a Batch Normalization layer, and a Rectified Linear Unit (ReLU) activation [28,29]. Conversely, the decoder symmetrically up-samples the feature maps using transposed convolutions to reconstruct the full-resolution output. The final layer of the network employs a Sigmoid activation function to produce the normalized, single-channel output mask.

The selection of the U-Net architecture, particularly its use of skip connections, was a deliberate choice critical to the success of this work. Simpler encoder–decoder structures often suffer from a significant loss of fine-grained spatial information during the down-sampling process. This information loss is especially detrimental for SRAF optimization, where the pixel-level precision of feature boundaries dictates the final lithographic performance. The U-Net’s skip connections directly counteract this issue by concatenating high-resolution feature maps from the encoder to the corresponding layers in the decoder. This mechanism ensures that the network, while learning the high-level contextual placement of SRAFs, never loses the critical, low-level information required to reconstruct their exact shapes and sharp edges. Furthermore, the final layer of the network employs a Sigmoid activation function. This is essential for mapping the network’s output to a normalized range of [0, 1], which serves as a continuous-tone representation (or probability map) of the final binary mask, making it directly suitable for the pixel-wise loss calculation.

### 3.3. Training Objective and Loss Function

The U-Net model is trained in a supervised manner. Its objective is to learn the mapping from a coarse SRAF input ($M_{\mathrm{coarse}}$) to its high-fidelity, LSM-optimized counterpart ($M_{\mathrm{LSM}}$). To achieve this, the model’s parameters are optimized to minimize the pixel-wise discrepancy between the predicted mask ($M_{\mathrm{pred}}$) and the ground-truth mask ($M_{\mathrm{LSM}}$).

**Figure 5:**
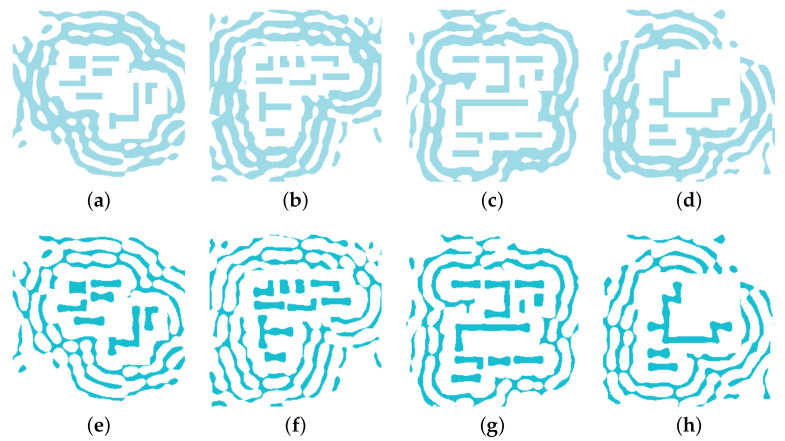
Examples of training data pairs. Panels (**a**–**d**) show four representative coarse SRAF input patterns (*x*). Panels (**e**–**h**) show the corresponding high-fidelity, LSM-optimized ground-truth layouts (*y*).

For this image-to-image regression task, we employ the Mean Squared Error (MSE) as the loss function. The MSE is defined as follows:

(12) $$L_{\mathrm{MSE}}=\frac{1}{N}\sum_{i=1}^{N}{\left(M_{\mathrm{LSM},i}-M_{\mathrm{pred},i}\right)}^{2},$$

where *N* is the total number of pixels in the layout, and $M_{\mathrm{LSM},i}$ and $M_{\mathrm{pred},i}$ represent the pixel values at a given location. Figure 5 provides a visual representation of this learning objective, illustrating the coarse SRAF inputs that the model receives and the corresponding high-fidelity targets that it learns to replicate. The training process iteratively adjusts the network’s parameters via backpropagation to minimize this loss, effectively teaching the model to reproduce the LSM-optimized SRAF patterns with high fidelity.

## 4. Experiment and Results

### 4.1. Experimental Setup

All simulations and model evaluations in this study were conducted within a consistently defined environment. The forward lithography process was modeled to represent a 193nm *ArF* immersion lithography system with a high numerical aperture (NA) of 1.35. The illumination source was a standard annular type with outer and inner partial coherence radii of $\sigma_{\mathrm{out}}=0.9$ and $\sigma_{\mathrm{in}}=0.6$, respectively. All mask patterns were discretized with a pixel resolution of 4 nm. For the wafer image simulation, we utilized the sigmoid-approximated CTR model with a steepness parameter a=85 and a normalized intensity threshold of $I_{\mathrm{th}}=0.18$.

The initial target patterns used to construct our dataset were sourced from the publicly available GAN-OPC [30] benchmark dataset. The complete dataset comprises 4847 unique layout patterns. Following the data generation pipeline described in Section 2, these patterns were processed and subsequently partitioned into a training set and a test set, containing 80% (3878 patterns) and 20% (970 patterns) of the data, respectively.

The core of our surrogate model is a U-Net architecture featuring a symmetric encoder-decoder path. The encoder consists of 8 downsampling blocks, with input channels progressing as [1, 8, 16, 32, 64, 128, 256, 512], where each block employs a stride of (2, 2). In total, the model comprises approximately 25.17 million trainable parameters. We trained the model for 100 epochs using the Adam optimizer with a batch size of 100. The initial learning rate was set to $1\times 10^{-3}$ and was decayed by a factor of 0.1 every 50 epochs.

To rigorously evaluate our framework, we employed distinct metrics for optimization and final model validation. As established in our methodology, PE serves as the primary cost function during the physics-based LSM optimization to generate the ground-truth data. For the final evaluation of our trained U-Net model, we utilize the suite of standard image comparison metrics previously mentioned, including Mean Squared Error (MSE), Mean Absolute Error (MAE), and Peak Signal-to-Noise Ratio (PSNR), to quantify the fidelity of the predicted masks against the ground truth. All computations were implemented in Python (v3.6.8) using the PyTorch (v1.10.1+cu102) framework and accelerated on an NVIDIA V100 GPU.

### 4.2. Comparative Analysis of Models

To empirically evaluate the proposed framework, a quantitative analysis was performed on the held-out test set. The performance of the U-Net model was benchmarked against three alternative deep learning architectures, as well as the initial coarse SRAF pattern, which served as a baseline. Table 1 summarizes the results across a suite of standard evaluation metrics.

The data presented in Table 1 indicates a clear hierarchy in model performance, with the U-Net architecture yielding the most accurate predictions. We primarily focus on Mean Squared Error (MSE) as a measure of pixel-wise fidelity and the Coefficient of Determination (R^2^) as an indicator of model fit. Our proposed U-Net achieved the lowest MSE (0.0182), a significant improvement over the other architectures, and an R^2^; score of 0.8916, indicating a strong correlation with the ground-truth data. In addition to its superior accuracy, the U-Net exhibits exceptional computational efficiency. Its training time of approximately 309 s is roughly 3.5 times faster than the ConvFNO and RFNO models, highlighting its advantageous balance of performance and resource cost.

**Figure 6:**
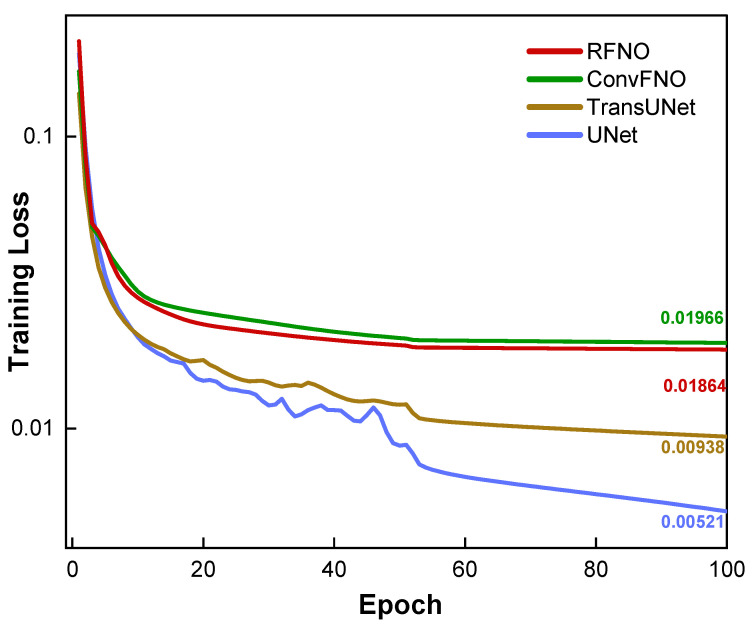
Training curve by different model.

The training dynamics were also investigated. Figure 6 plots the training loss (MSE) as a function of the training epoch. As illustrated, all four models exhibit stable convergence over the 100 epochs, indicating that each architecture was capable of effectively learning the SRAF refinement task from the dataset. However, a clear distinction in final performance is evident from the curves. The U-Net models converge to a significantly lower final loss value than the RFNO, ConvFNO and U-Net Transformer models. This observation from the training dynamics is in strong agreement with the superior accuracy metrics for the U-Net reported in Table 1, and it further validates that the U-Net-based architecture provides a better fit for this specific optimization problem.

**Figure 7:**
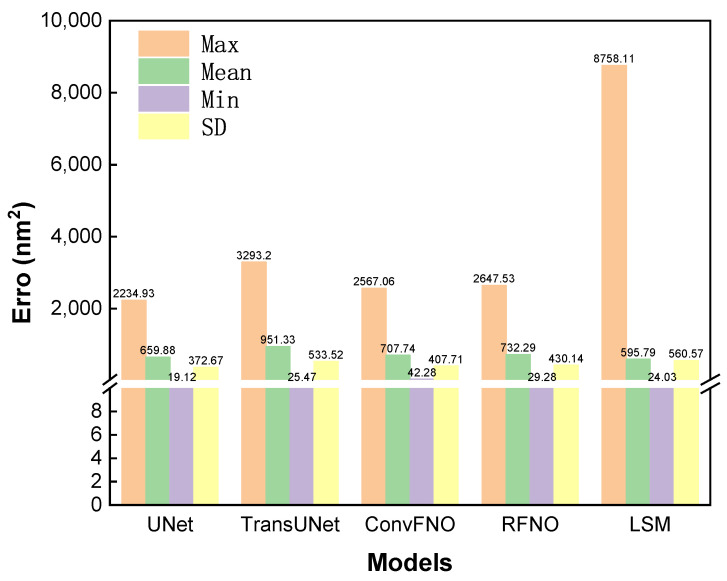
Comparison of PE statistics for the final layouts generated by the four deep learning models and the LSM ground-truth generation method.

To provide a more intuitive comparison of the critical PE metric, Figure 7 presents a bar chart visualizing the mean, maximum, and minimum PE values for the final layouts produced by each method. The chart confirms that the U-Net is the top-performing deep learning architecture, achieving the lowest values across all three PE statistics. When benchmarked against the iterative LSM baseline, our U-Net model achieves a highly comparable mean PE of 659.88, which is close to the LSM’s result of 595.79. The primary advantage of our data-driven approach is its ability to mitigate worst-case scenarios by significantly reducing the maximum PE compared to the LSM baseline (2234.93 vs. 8758.11). This enhanced robustness is further corroborated by the standard deviation (SD) of the PE across the test set: the U-Net’s SD was 372, significantly lower than the LSM’s 560, indicating a more stable and predictable performance from our model. While these PE statistics capture numerical accuracy, the outputs of the other three models exhibited qualitative instabilities, the differences of which will be demonstrated in the subsequent qualitative analysis.

### 4.3. U-Net Model Validation

**Figure 8:**
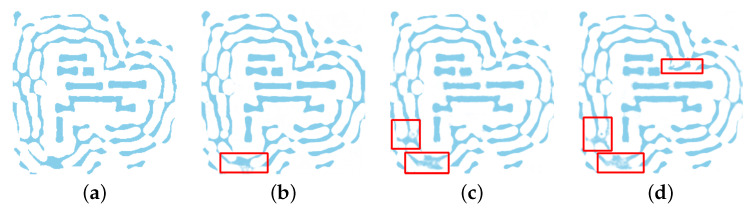
Qualitative comparison of the SRAF layouts predicted by the four evaluated models: (**a**) proposed UNet; (**b**) transformer UNet; (**c**) RFNO; and (**d**) ConvFNO. Note the presence of non-binary artifacts in (**b**–**d**), while the output from our proposed U-Net (**a**) is clean and binary-like. The red boxes highlight areas exhibiting these non-binary, “shadowing” artifacts or inconsistencies in the predicted SRAF patterns.

A qualitative analysis was performed on the test set to visually compare the performance of the different deep learning architectures. A critical requirement for any SRAF generation method is the ability to produce clean, binary masks suitable for manufacturing. As illustrated in the detailed view in Figure 8, the four evaluated models show significant differences in this regard. The outputs from the UNet Transformer, RFNO, and ConvFNO all exhibit non-binary, grayscale artifacts (“shadowing”), where many pixels converge to intermediate values. These artifacts render their predicted masks physically unrealizable. In stark contrast, the output from our proposed U-Net is clean and distinctly binary, establishing it as the only architecture among those tested that produces a qualitatively viable solution.

Having identified the U-Net as the superior model, we performed a more detailed visual validation of its refinement capability. The performance of the optimized U-Net model was validated against the LSM-generated ground truth through both qualitative and quantitative assessments.

**Figure 9:**
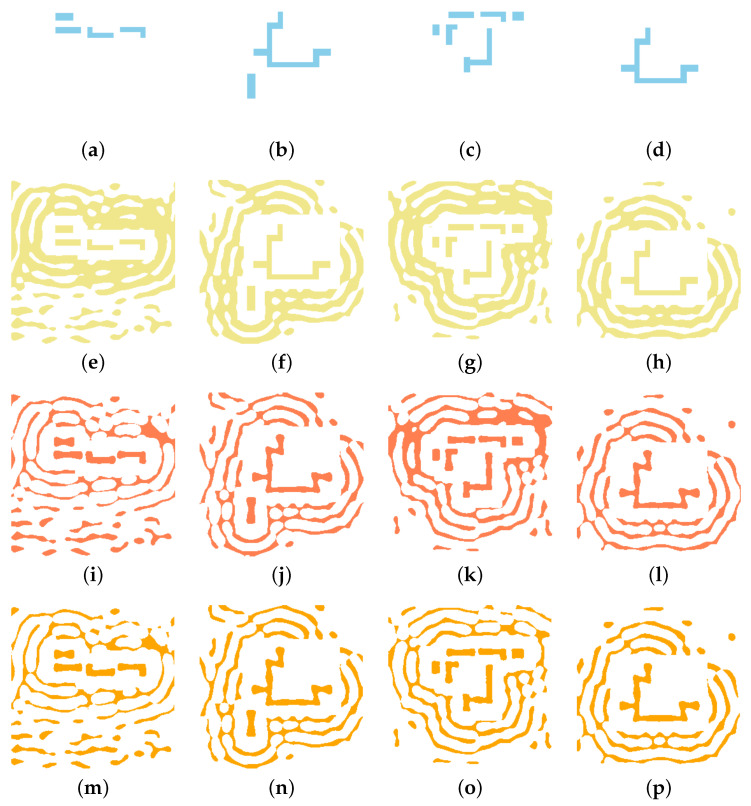
Visual validation of the U-Net model on four test patterns. For each case (column), the rows display the following: (**a**–**d**) the initial target pattern; (**e**–**h**) the coarse SRAF input (*x*); (**i**–**l**) the LSM-generated ground truth (*y*); and (**m**–**p**) our U-Net’s final prediction ($\hat{y}$).

A visual comparison is provided in Figure 9, which displays representative layouts from the test set. The qualitative results indicate a high degree of pattern fidelity between the U-Net’s prediction and the ground truth. The model effectively removes the topological artifacts present in the coarse input and accurately reproduces the complex, optimized geometries generated by the LSM.

For a rigorous quantitative evaluation, the final PE was calculated for 16 representative cases selected from the test set, with the results summarized in Table 2. The data demonstrates our model’s dual advantages of high predictive speed and accuracy. While the iterative LSM requires significant computation time per pattern, our trained U-Net produces a refined layout via a single, rapid inference pass.

For a rigorous quantitative validation of our model’s accuracy, Table 2 presents a case-by-case comparison of the final Pattern Error (PE) for 16 representative layouts selected from the test set. The results indicate that the final accuracy of our proposed U-Net model is highly comparable to that of the iterative LSM. Across this diverse subset of cases, our model achieved an average PE of 538.60, closely approaching the LSM’s average of 549.24. While performance on individual layouts varies—with our model demonstrating superior accuracy in the majority of cases (e.g., Case 3, Case 8) and the LSM performing marginally better in others (e.g., Case 5, Case 9)—the key finding is the consistent, high-fidelity performance of our data-driven approach. This near-equivalent accuracy is achieved via a single, rapid inference pass, in stark contrast to the computationally prohibitive iterative process required by the LSM. Therefore, our framework is validated as a solution that successfully resolves the long-standing trade-off between computational speed and pattern fidelity in SRAF optimization.

### 4.4. Computational Performance Analysis

To provide a detailed and transparent comparison of computational performance, Table 3 displays a breakdown of the online processing times. The traditional CPU-based LSM serves as a baseline, requiring 140.78 s to converge for a single layout.

For modern GPU-based approaches, the workflow begins with a preprocessing step to generate a coarse SRAF layout, which takes approximately 0.65 s. This coarse layout is a common prerequisite for both the accelerated iterative algorithm and our U-Net model. Following this shared step, the subsequent refinement processes differ significantly. The GPU-accelerated LSM requires an additional 0.58 s for iterative optimization. In contrast, our U-Net performs the refinement in a single inference pass of just 0.05 s.

Therefore, the core refinement step in our data-driven approach is over 11 times faster (0.58 s vs. 0.02 s) than the GPU-accelerated iterative method. This substantial efficiency gain, combined with the multi-order-of-magnitude speedup over the CPU-based process, confirms that our framework offers a solution for accelerating the SRAF synthesis workflow.

## 5. Discussion and Conclusions

The experimental results of this study demonstrate that a U-Net-based surrogate model can serve as a highly efficient and effective solution for SRAF optimization, successfully overcoming the persistent trade-off between accuracy and computational cost that constrains conventional methodologies. The superiority of the U-Net architecture is attributed to its suitability for this specific physical problem. The U-Net’s distinctive skip connections provide a direct pathway for fusing high-level contextual information from its encoder with fine-grained spatial details in its decoder, a mechanism that is exceptionally effective at preserving the precise boundary information critical for high-fidelity SRAF reconstruction. Perhaps the most significant finding is the model’s ability to generate layouts with a measurably lower PE than the LSM-generated data that it was trained on. This outperformance is particularly evident in complex layouts, where the model proves more robust at mitigating the large, outlier errors (maximum PE) to which the iterative LSM is occasionally susceptible. This suggests the U-Net is not merely an approximator but a more effective optimization engine, capable of generalizing from the training data to discover solutions in the high-dimensional space that are potentially more globally optimal than those found by the iterative, path-dependent LSM.

Beyond its function as a standalone surrogate, the U-Net model also shows significant potential as an effective initializer for a hybrid optimization strategy, a concept quantified in Table 4. The data reveals that, by taking the U-Net’s high-fidelity prediction as an advanced starting point and applying just 10 subsequent LSM iterations, the final average PE is reduced to 504.59. This result is approximately 15% more accurate than the original, computationally expensive LSM (Mean PE: 595.79). This improved accuracy is achieved with a minimal increase in computational time; the total online time for the hybrid method is just 0.73 s, representing a ∼190-fold acceleration compared to the 140.78 s conventional LSM process. This synergy between a rapid, data-driven prediction and a final, physics-based refinement highlights a practical workflow for achieving a higher final accuracy while still maintaining a substantial reduction in computational time.

The principal contribution of this study was to validate a new surrogate modeling framework, and our focus on PE as the primary metric was a deliberate choice for methodological consistency. PE was the explicit optimization objective of the physics-based LSM used to generate our ground-truth data, and the U-Net’s task was to learn a surrogate for this specific, PE-driven process. Therefore, PE is the most direct and scientifically valid metric to assess our model’s success. Adopting a different primary metric (like EPE) would represent a reconstruction of the entire research, as it would first require redesigning the core physics-based optimizer itself. Besides, SRAF placement usually preludes OPC where the performance is evaluated with designed metrics in its own significance, more often than not, other than EPE.

Having established this foundational proof-of-concept for high-speed SRAF placement, the logical next phase is to investigate robustness on wider, out-of-distribution (OOD) datasets. Our future work will then focus on integrating this upstream SRAF placement engine with a downstream, EPE-driven module. This is the appropriate stage to incorporate a full suite of metrics, including an EPE-driven OPC engine and a differentiable penalty function for manufacturing rule constraint (MRC) compliance. Integrating these sequential stages is the final step toward a completely automated and highly efficient mask synthesis workflow.

## Figures and Tables

**Table 1:** Performance comparison of different deep learning architectures.

	UNet	TransUNet	RFNO	ConvFNO
MSE	0.0182	0.0226	0.0197	0.0205
RMSE	0.1348	0.1503	0.1402	0.1432
MAE	0.0316	0.0370	0.0385	0.0406
PSNR	17.41	16.46	17.07	16.88
R^2^	0.8916	0.8653	0.8828	0.8776
Training Time (s)	309.0682	321.4919	1119.6746	1040.8462
Prediction Time (s)	1.01	1.65	1.92	1.71

**Table 2:** Performance comparison of the proposed method and the LSM.

Benchmarks	Level-Set Method (PE) (nm^2^)	Ours (PE) (nm^2^)
Case 1	619.18	586.30
Case 2	175.72	132.05
Case 3	801.50	642.09
Case 4	336.32	337.32
Case 5	610.59	633.59
Case 6	581.88	543.82
Case 7	656.89	586.41
Case 8	1558.45	1424.75
Case 9	1199.43	1248.10
Case 10	573.24	491.65
Case 11	538.67	532.07
Case 12	544.47	473.18
Case 13	634.87	623.93
Case 14	368.58	368.87
Case 15	42.02	36.98
Case 16	545.99	506.53
Average	549.24	538.60

**Table 3:** Breakdown and comparison of online computational time per ayout.

Method	Platform	CTM (s)	Refinement (s)	Total Time (s)
Conventional LSM	CPU	68.98	71.80	140.78
GPU-Accelerated LSM	GPU	0.65	0.58	1.23
Proposed U-Net	GPU	0.65	0.02	0.67

**Table 4:** Performance comparison of the hybrid optimization strategy.

Method	Mean (PE) (nm^2^)	Max (PE) (nm^2^)	Min (PE) (nm^2^)	Times (s)
Conventional LSM	595.79	8758.11	24.03	140.78
Proposed U-Net	659.88	2234.93	19.12	0.67
Hybrid (U-Net + 10 LSM)	504.59	1703.34	20.14	0.73

## Data Availability

The original contributions presented in this study are included in the article, and further inquiries can be directed to the corresponding author.
